# Antibacterial Role of SO_4_^2-^, NO_3_^-^, C_2_O_4_^2-^ and CH_3_CO_2_^-^ Anions on Cu(II) and Zn(II) Complexes of a Thiadiazole-derived Pyrrolyl Schiff Base

**DOI:** 10.1155/MBD.2002.263

**Published:** 2002

**Authors:** Zahid H. Chohan, Humayun Pervez, Abdul Rauf, Claudiu T. Supuran

**Affiliations:** 1 Department of Chemistry Islamia University Bahawalpur Pakistan; 2 Department of Chemistry Bahauddin Zakariya University Multan Pakistan; 3 University of Florence Dipartimento di Chimica Laboratorio di Chimica Bioinorganica Via della Lastruccia 3, Rm 188, Polo Scientifico Firenze 50019-Sesto Fiorentino Italy

## Abstract

A condensation reaction of 2-amino-1,3,4-thiadiazole with 2-pyrrolecarboxaldehyde to form tridentate NNN donor Schiff base has been performed. The prepared Schiff base was further used for the formation of metal complexes having stoichiometry [M(L)_2_]X_n_, where M=Cu(II) or Zn(II), L=N-(2-pyrrolylmethylene)-2-amino-1,3,4-thiadiazole, X=SO_4_^2−^, NO_3_^−^, C_2_O_4_^2−^ or CH_3_CO_2−_ and n=1 or 2. The new compounds described here have been characterized by their physical, spectral and analytical data, and have been screened against several bacterial strains such as *Escherichia coli*, *Staphylococcus aureus*, and *Pseudomonas aeruginosa*. The antibacterial potency of the Schiff base increased upon chelation/complexation, having the same metal ion (cation) but different anions opening up a novel approach in finding new ways to fight against antibiotic resistant strains.


### ANTIBACTERIAL ROLE OF $SO_4^{2-}$, $NO_3^-$, $C_2O_4^{2-}$ AND $CH_3CO_2^-$ ANIONS ON Cu(II) AND Zn(II) COMPLEXES OF A THIADIAZOLE-DERIVED PYRROLYL SCHIFF BASE

Zahid H. Chohan*[1], Humayun Pervez[2], Abdul Rauf[1] and Claudiu T. Supuran[3]

[1]Department of Chemistry, Islamia University, Bahawalpur, Pakistan
[2]Department of Chemistry, Bahauddin Zakariya University, Multan, Pakistan
[3] University of Florence, Dipartimento di Chimica, Laboratorio di Chimica Bioinorganica, Via della Lastruccia 3, Rm 188, Polo Scientifico, 50019-Sesto Fiorentino Firenze, Italy

### ABSTRACT
A condensation reaction of 2-amino-1,3,4-thiadiazole with 2-pyrrolecarboxaldehyde to form tridentate NNN donor Schiff base has been performed. The prepared Schiff base was further used for the formation of metal complexes having stoichiometry $[M(L)_2]X_n$, where M=Cu(II) or Zn(II), L=N-(2-pyrrolylmethylene)-2-amino-1,3,4-thiadiazole, X=$SO_4^{2-}$, $NO_3^-$, $C_2O_4^{2-}$ or $CH_3CO_2^-$ and n=1 or 2. The new compounds described here have been characterized by their physical, spectral and analytical data, and have been screened against several bacterial strains such as *Escherichia coli, Staphylococcus aureus*, and *Pseudomonas aeruginosa.* The antibacterial potency of the Schiff base increased upon chelation/complexation, having the same metal ion (cation) but different anions opening up a novel approach in finding new ways to fight against antibiotic resistant strains.

### INTRODUCTION
Thiadiazoles are largely used as antibacterial[1], antifungal[2], antitumor[3,4], diuretic[5,6], antiepileptic[7], antiulcer[8] and antileukemia[9,10] agents. These compounds possess such interesting biological properties that may be conferred to them by their strong aromatic ring system[11]. As ligands, they also provide many potential binding sites for complexation and have obtained a diversified biological activity[12-14] by the result of such chelation. It has been suggested[16] that the interaction of the metal ion with potentially biologically active compounds lead to destroy or greatly help to diminish[17-19] the role of bacteria-/virus-/cancer-associated organisms and therefore, represent a significant route for designing and establishing novel metal-based antibacterial, antiviral and anticancer therapies[20]. In previous works we have explored the biological properties of thiadiazole-derived compounds and the effect of chelation on their bactericidal properties[21]. In order to evaluate the role of anions staying outside of the coordination sphere of the metal chelate, we report here metal chelates of the already reported[21] Schiff base, obtained by the condensation of 2-amino-1,3,4-thiadiazole with 2-pyrrolcarboxaldehyde to form tridentate NNN donor ligand, i.e., N-(2-pyrrolylmethylene)-2-amino-1,3,4-thiadiazole (L)' having the same metal ion (cation) but different anions such as, sulfate, nitrate, acetate and oxalate of the type $[M(L)_2]X_n$ where M=Cu(II) or Zn(II), L= N-(2-pyrrolylmethylene)-2-amino-1,3,4-thiadiazole (Fig. 1), X=$SO_4^{2-}$, $NO_3^-$, $C_2O_4^{2-}$ or $CH_3CO_2^-$ and n=1 or 2. These compounds have been characterized and screened for their antibacterial activity against pathogenic strains of *Escherichia coli, Staphylococcus aureus*, and *Pseudomonas aeruginosa.*

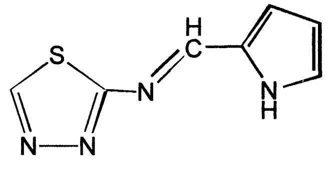

Fig.1: Structure of the Schiff base (L)

### EXPERIMENTAL
### Material and Methods
All chemicals and solvents used in syntheses were of Analar grade purity. IR spectra were recorded on a Philips Analytical PU 9800 FTIR spectrophotometer as KBr discs. UV-Visible spectra were obtained in DMF on a Hitachi U-2000 double-beam spectrophotometer. C, H and N analyses was carried out by Butterworth Laboratories Ltd. Conductance of the metal complexes was determined in DMF on a Hitachi YSI-32 model conductometer. Magnetic measurements were made on solid complexes using the Gouy method. Melting points were recorded on a Gallenkamp apparatus and are uncorrected.

**Preparation of the Metal(II) Complexes**

A warm ethanol solution (20 mL) of the Schiff base (0.002 M) was added to a magnetically stirred solution of the respective metal(II) salt (0.001 M) in distilled water (25 mL). The mixture was refluxed for 1 h and cooled thereafter to room temperature. On cooling, a precipitate was formed which was filtered, washed with ethanol, acetone and ether, and dried by suction. Crystallization from aqueous ethanol (70:30) gave the desired metal complex. All other metal derivatives were obtained following the same method.

**Antibacterial Studies**

The synthesized metal complexes, as well as the uncomplexed Schiff base were screened for their antibacterial activity against pathogenic bacterial strains of *Escherichia coli, Staphylococcus aureus* and *Pseudomonas aeruginosa*. The compounds were tested at a concentration of 30 µg/0.01 mL in DMF solution using the paper disc diffusion method as reported as earlier[33-37]. The diameter of the susceptibility zones was measured in mm. The measured susceptibility zones were the clear zones around the discs killing the bacteria.

Table 1. Physical and Analytical Data of the Metal(II) Chelates prepared in this study.

| No | Metal chelate/ Mol. Formula | Yield (%) | M.p (°C) (decomp) | B.M. ($\mu_{eff}$) | Calc (Found)% C H N |
|---|---|---|---|---|---|
| 1 | $[Cu(L)_2]SO_4$ [515.5] $C_{14}H_{12}CuN_8O_4S_3$ | 60 | 218-220 | 1.7 | 32.6 2.3 21.7 (32.9) (2.1) (21.5) |
| 2 | $[Cu(L)_2](NO_3)_2$ [543.5] $C_{14}H_{12}CuN_{10}O_6S_2$ | 62 | 211-213 | 1.6 | 30.9 .2.2 25.8 (31.2) (2.4) (25.7) |
| 3 | $[Cu(L)_2](C_2O_4)$ [507.5] $C_{16}H_{12}CuN_8O_4S_2$ | 61 | 209-211 | 1.5 | 37.8 2.4 22.1 (37.5) (2.0) (22.6) |
| 4 | $[Cu(L)_2]CH_3CO_2$ [537.5] $C_{18}H_{18}CuN_8O_4S_2$ | 63 | 215-217 | 1.5 | 40.2 3.3 20.8 (40.6) (3.1) (20.5) |
| 5 | $[Zu(L)_2]SO_4$ [517.4] $C_{14}H_{12}ZnN_8O_4S_3$ | 62 | 205-207 | Dia | 32.5 2.3 21.6 (32.2) (2.7) (21.3) |
| 6 | $[Zn(L)_2](NO_3)_2$ [545.4] $C_{14}H_{12}ZnN_{10}O_6S_2$ | 62 | 214-216 | Dia | 30.8 2.2 25.7 (30.5) (2.6) (25.4) |
| 7 | $[Zn(L)_2](C_2O_4)$ [509.4] $C_{16}H_{12}ZnN_8O_4S_2$ | 60 | 212-214 | Dia | 37.7 2.4 22.0 (38.1) (2.7) (21.8) |
| 8 | $[Zn(L)_2]CH_3CO_2$ [539.4] $C_{18}H_{18}ZnN_8O_4S_2$ | 61 | 216-218 | Dia | 40.0 3.3 20.8 (40.3) (3.7) (20.5) |

### RESULTS AND DISCUSSION

**Physical Properties**

The Schiff base (Fig. 1) was prepared by refluxing an appropriate amount of 2-amino-1,3,4-thiadiazole with 2-pyrrolcarboxaldehyde in ethanol in a 1 : 1 molar ratio. The structure of the Schiff base was established as reported[21] earlier. All metal complexes (1-8) (Table 1) were air stable and prepared by the stoichiometric reaction of the corresponding metal(II) salts with the Schiff base ligand, in a molar ratio M:L of 1:2. These complexes are intensely colored and amorphous solids, which decompose without melting. They are insoluble in common organic solvents such as ethanol, methanol, chloroform or acetone being only soluble in DMSO and DMF. Molar conductance values of the soluble complexes in DMF show their values (132-147 $ohm^{-1}cm^2mol^{-1}$) indicating[22] that they are all electrolytic in nature.

**IR Spectra**

IR spectra of the reported Schiff base showed the absence of bands at 1735 and 3420 $cm^{-1}$ due to carbonyl ν(C=O) and ν($NH_2$) stretching vibrations (present in the starting materials). Instead a strong new band at ~1640 $cm^{-1}$ assigned as azomethine ν(HC=N) vibration appeared[23] indicating condensation of the two moieties. The comparison of the infrared spectra of the Schiff base with their metal chelates indicated that the Schiff base was basically coordinated to the metal ions tridentately. The band appearing at 1640 $cm^{-1}$ due to the azomethine vibration is shifted to lower frequency by ~15-20 $cm^{-1}$ indicating[24] the participation of the azomethine nitrogen in complexation. The band at 1620 $cm^{-1}$ assigned to the thiadiazole ring ν(C=N) vibrations is also shifted to lower frequency by ~15 $cm^{-1}$ which is indicative[24] of the involvement of the thiadiazole ring nitrogen in chelation. Further evidence of the coordination of the Schiff base with the metal ions was shown by the appearance of a weak low frequency new band at 525-530 $cm^1$ (Table 2). This was, in turn, assigned[25] to ν(M-N). This new band was observable only in the spectra of the metal complexes and not in the spectra of the uncomplexed Schiff base and thus confirm the participation of the nitrogen heteroatom in the coordination.

Table 2. IR and UV-Visible Spectra Data of the Metal(II) Chelates.

| No | IR ($cm^{-1}$) | $\lambda_{max}$ ($cm^{-1}$) |
|---|---|---|
| 1 | 1625 (HC=N), 1605 (C=N), 525 (M-N). | 22,155, 30,140 |
| 2 | 1620 (HC=N), 1605 (C=N), 525 (M-N). | 22,265, 30,265 |
| 3 | 1620 (HC=N), 1605 (C=N), 530 (M-N). | 22,275, 30,375 |
| 4 | 1625 (HC=N), 1610 (C=N), 530 (M-N). | 22,270, 30,250 |
| 5 | 1620 (HC=N), 1610 (C=N), 525 (M-N). | 28,665 |
| 6 | 1620 (HC=N), 1605 (C=N), 530 (M-N). | 28,745 |
| 7 | 1625 (HC=N), 1605 (C=N), 525 (M-N). | 28,850 |
| 8 | 1625 (HC=N), 1610 (C=N), 530 (M-N). | 28,785 |

**NMR Spectra**

The NMR spectral data of the Schiff base as well as some of its Zn(II) complexes as chloride taken in DMSO-$d_6$ is reported earlier[21]. The Schiff base exhibited signals due to all the expected protons in their expected region and has been fully identified from the integration curves found to be equivalent to the total number of protons deduced from the proposed structure. These were compared with the reported[26] signals of known structurally related compounds and give further support for the composition of this new Schiff base as well as its complexes suggested by the IR and elemental analyses data. Comparison of the chemical shifts of the uncomplexed Schiff base with those of the corresponding zinc(II) complexes show that some of the resonance are shifted upon complexation. In each case, the protons assigned due to the heteroaromatic (C=N) ring and azomethine (HC=N) were found[21] at around $\delta$ 7.3-8.8 ppm in the spectra of the Schiff base. These protons undergo downfield shift in the zinc complexes indicating participation of these groups in coordination with the metal ions. The same shifts were observed in the $^{13}C$ NMR spectral data of the Schiff base versus their zinc complexes.

**Magnetic Moment and Electronic Spectra**

The room temperature magnetic moment of the solid copper(II) complexes having tetrahedral or octahedral geometry normally exhibits magnetic moments in the range 1.6-1.8 B.M. The magnetic moment of the present Cu(II) complexes (Table 1) was found to lie in the range of 1.5-1.7 B.M, indicative[27,28] of one unpaired electron per Cu(II) ion, consistent with its distorted octahedral geometry. The Zn(II) complexes were all found diamagnetic. The electronic spectra of the Cu(II) chelates (Table 2) showed absorption bands between 10 Dq for a distorted octahedral geometry (Fig 2) corresponding[29,30] to the transitions $^2E_g \rightarrow {}^2T_{2g}$. The bands observed at 22155-22275 $cm^{-1}$ and 30,140-30,375 $cm^{-1}$ may be assigned[31] to intra-ligand charge transfer transitions. The diamagnetic Zn(II) complexes did not show any d-d bands and their spectra are dominated by charge transfer bands. The charge transfer band at 28665-28850 $cm^{-1}$ was assigned[32] to $^2E_g \rightarrow {}^2T_{2g}$ transitions possibly in an octahedral environment (Fig 2).

Table 3. Antibacterial Activity Data of the Schiff base and its Metal(II) chelates 1-8.

| Schiff base/ Chelate | Microbial Species | | |
|---|---|---|---|
| | a | b | c |
| L | ++ | ++ | + |
| 1 | +++ | ++ | ++ |
| 2 | +++ | ++++ | ++ |
| 3 | +++ | +++ | +++ |
| 4 | ++++ | +++ | +++ |
| 5 | ++++ | ++++ | ++ |
| 6 | ++++ | +++ | +++ |
| 7 | +++ | ++++ | +++ |
| 8 | +++ | ++++ | +++ |

a= *Escherichia coli*, b= *Staphylococcus aureus*, c= *Pseudomonas aeruginosa*
Inhibition zone diameter mm (% inhibition): +, 6-10 (27-45 %); ++, 10-14 (45-64 %); +++, 14-18 (64-82 %); ++++, 18-22 (82-100 %). Percent inhibition values are relative to inhibition zone (22 mm) of the most active compound with 100 % inhibition.

The Schiff base and its complexes individually exhibited varying degrees of inhibitory effects on the growth of the tested bacterial species (Table 3). The antibacterial results evidently show that the activity of the Schiff base became more pronounced and significant when coordinated to these metal ions. It was observed that when the same metal chelate having different anions was individually screened, the degree of bactericidal activity also varied. For example, the Cu(II) complex having nitrate as anion was more bactericidal than the Cu(II) complex with sulfate, oxalate or acetate anions. Similarly, the Cu(II) complex oxalate was more antibacterial than the complex having acetate, chloride or sulfate. Similar results were found in the case of Zn(II) complexes. It is observed that the order of potency, in comparison to the metal complexes having chloride anions evaluated and reported[21] earlier, is

$$NO_3^- > C_2O_4^{2-} > CH_3CO_2^- > Cl^- > SO_4^{2-}$$

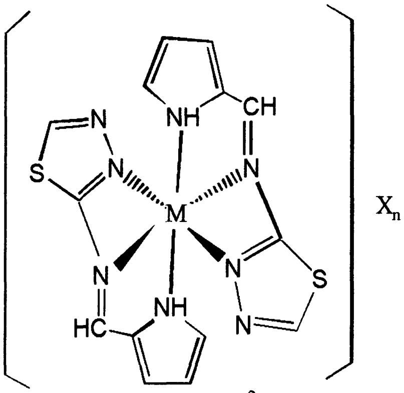

$X = NO_3^- > C_2O_4^{2-} > CH_3CO_2^- > Cl^- > SO_4^{2-}$, M = Cu(II) or Zn(II), n = 1 or 2

Fig. 2: Proposed Structure of the Metal(II) Complexes **1-8**.

On the basis of these observations, it is claimed that the process of chelation dominantly affects the biological behavior of the compounds that are potent against some bacterial strains. From these studies, it is also proposed that different anions do play a significant role in the biological behavior of the metal chelates. It is suspected that factors such as solubility, different dipole moment and cell permeability mechanisms may be influenced by the presence of the different anions which in turn, affect the overall mechanism of permeation through the lipoid layer of the organisms thus killing them more effectively and efficiently.

### ACKNOWLEDGEMENT

The authors gratefully acknowledge the help of the Department of Pathology, Qaid-e-Azam Medical College, Bahawalpur, in undertaking the antibacterial studies.

### REFERENCES

1. a) Z. H. Chohan, M. F. Jaffery and C. T. Supuran, *Metal-Based Drugs*, **8**, 95 (2001). b) M. Barboui, M. Cimpoesu, C. Guran and C. T. Supuran, *Metal-Based Drugs*, **3**, 227 (1996). c) E. Profpt, H. Teubner and W. Weuffen, *Arch. Exp. Veterinaermed*, **21**, 225 (1967).
2. a) A. S. Yani, *J. Ind. Chem*, **68**, 529 (1991). b) A. Mastrolorenzo and C. T. Supuran, *Metal-Based Drugs*, **7**, 49 (2000). c) A. Mastrolorenzo, A. S cozzafava and C. T. Supuran, *Eur. J. Pharmaceut. Sci*, **11**, 99 (2000). d) A. Scozzafava and C. T. Supuran, *J. Enz. Inhib*, **15**, 517 (2000). e) O. T. Savulescu, V. Barbu and V. Tudoescu, *Phytopathology*, **59**, 129 (1969).
3. M. Miyamoto, R. Koshiura, H. Yokoi, C. Mori, T. Hasegawa and K. Takotri, *Chem. Pharm. Bull*, **33**, 5126 (1985).
4. a) N. H. Jayaram, H. Zhen, E. N. Weining and M. Kamran, *Cancer. Res*, **53**, 2344 (1995). b) T. H. Maren, *J. Glaucoma*, **4**, 49 (1995).
5. T. H. Maren, *Physiol. Rev*, **47**, 595 (1967).
6. T. H. Maren, B. W. Clare and C. T. Supuran, Roum. *Chem. Quart. Rev*, **2**, 259 (1994).
7. D. M. Woodbury, "*Antiepileptic Drugs*: Mechanism of Action", Raven Press, New York (1980).
8. I. Puscas and C. T. Supuran, "*Farmacologia Clinica da Ulcer Peptica*", Rio de Janeiro (1996).

9. K. Y. R. Zee-Cheng and C. C. Cheng, *J. Med. Chem*, **28**, 1216 (1985).
10. S. C. Sharma, *Bull. Chem. Soc. Jpn*, **40**, 2422 (1967).
11. G. Kornis, "*1,3,4-Thiadiazoles in Comprehensive Heterocyclic Chemistry*", Ed, A. R. Katritzky, Pergamon, New York, Vol 6, part 4B, pp. 545-578 (1984).
12. E. S. Raper, *Coord. Chem. Rev*, **129**, 91 (1994).
13. G. Alzuet, G. Casanova, J. A. Ramirez, J. Borass and O. Carugo, *J. Inorg. Biochem*, **57**, 219 (1995).
14. G. Alzuet, L. Casella, A. Perotti and J. Borras, *J. Chem. Soc (Dalton Trans),* 2347 (1994).
15. D. K. Johnson, T. B. Murphy, T. B. Rose, W. H. Goodwin, and L. Pickart, *Inorg. Chim. Acta,* 67, 159 (1982).
16. S. Kirschner, Y. K. Wei, D. Francis and D. Bergman, *J. Med. Chem*, **9**, 369 (1966).
17. W. Beerheide, M. M. Sim, Y. J. Tan, H. U. Bernard and A. E. Ting, *Bioorg. Med. Chem*, **8**, 2549 (2000).
18. S. E. Livingstone, J. D. Nolan and A. E. Mihkelson, *Inorg. Chem*, **9**, 2545 (1970).
19. M. Huang, A. Maynard, J. A. Turpin, L. Graham, G. M. Janini, D. G. Covell and W. G. Rice, *J. Med. Chem,* **41**, 1371 (1998).
20. M. E. Heim, "*Metal Complexes in Chemotherapy*", Verlag Chemie, Weinheim (1993).
21. Zahid H. Chohan, Humayun Pervez, Abdul Rauf, Andrea Scozzafava, and Claudiu T. Supuran, *J. Enz. Inhib* (in press).
22. a) A. M. Shallary, M. M. Moustafa and M. M. Bekheit, *J. Inorg. Nucl. Chem*, **41**, 267 (1979). b) W. J. Geary, *Coord. Chem. Rev*, 7, 81 (1971).
23. L. J. Bellamy, "*The Infrared Spectra of Complex Molecules*", 3rd Ed, Methuen, London (1966).
24. M. Yongxiang, Z. Zhengzhi, M. Yun and Z. Gang, *Inorg. Chim. Acta*, **165**, 185 (1989).
25. K. Nakamoto, "*Infrared Spectra of Inorganic and Coordination Compounds*", 2nd Ed, Wiley Interscience, New York (1970).
26. D. H. Williams and I. Fleming, "*Spectroscopic Methods in Organic Chemistry*", 4th Ed, Mc Graw Hill, London (1989).
27. D. W. Meek, R. S. Drago, and T. S. Piper, *Inorg. Chem*, **1**, 285 (1962).
28. R. S. Drago, "*Physical Methods in Inorganic Chemistry*", Reinhold, New York (1965).
29. B. N. Figgis, "*Introduction to Ligand Fields*", J. Wiley, New York (1976).
30. A. B. P. Lever, "*Inorganic Electronic Spectroscopy*", Elsevier, Amsterdam (1984).
31. T. M. Dunn, J. Lewis and R. C. Wilkins, "*The Visible and Ultraviolet Spectra of Complex Compounds in Modern Coordination Chemistry*", Interscience, New York (1960).
32. R. L. Carlin, "*Transition Metal Chemistry*", Vol 1, Marcel Decker, New York (1965).
33. Zahid H. Chohan and Sabuhi Mushtaq, *Pak. J. Pharmaceut. Sci*, **13**, 21 (2000).
34. Zahid H. Chohan, *Synth. React. Inorg. Met-Org. Chem*, **31**, 1 (2001).
35. Zahid H. Chohan and Claudiu T. Supuran, *Main Group Met. Chem.*, **24**, 399 (2001).
36. Zahid H. Chohan, Asifa Munawar and Claudiu T. Supuran, *Metal-Based Drugs*, **8**, 137 (2001).
37. Zahid H. Chohan, M. A. Farooq and Claudiu T. Supuran, *Metal-Based Drugs*, **8**, 171 (2001).

**Received: February 8, 2002 – Accepted: February 19, 2002 – Accepted in publishable format: February 22, 2002**

